# Injury patterns of the acromioclavicular ligament complex in acute acromioclavicular joint dislocations: a cross-sectional, fundamental study

**DOI:** 10.1186/s12891-016-1240-3

**Published:** 2016-09-06

**Authors:** Dirk Maier, Martin Jaeger, Kilian Reising, Matthias J. Feucht, Norbert P. Südkamp, Kaywan Izadpanah

**Affiliations:** Department of Orthopedics and Trauma Surgery, University Medical Center Freiburg, Hugstetter Strasse 55, 79106 Freiburg, Germany

**Keywords:** Acromioclavicular joint, Acute dislocation, Acromioclavicular ligament complex, Articular disc, Deltoid-trapezoidal fascia, Acromioclavicular ligament repair, Horizontal instability

## Abstract

**Background:**

Horizontal instability impairs clinical outcome following acute acromioclavicular joint (ACJ) reconstruction and may be caused by insufficient healing of the superior acromioclavicular ligament complex (ACLC). However, characteristics of acute ACLC injuries are poorly understood so far. Purposes of this study were to identify different ACLC tear types, assess type-specific prevalence and determine influencing cofactors.

**Methods:**

This prospective, cross-sectional study comprised 65 patients with acute-traumatic Rockwood-5 (*n* = 57) and Rockwood-4 (*n* = 8) injuries treated operatively by means of mini-open ACJ reduction and hook plate stabilization. Mean age at surgery was 38.2 years (range, 19–57 years). Standardized pre- and intraoperative evaluation included assessment of ACLC tear patterns and cofactors related to the articular disc, the deltoid-trapezoidal (DT) fascia and bony ACJ morphology. Articular disc size was quantified as 0 = absent, 1 = remnant, 2 = meniscoid and 3 = complete.

**Results:**

All patients showed complete ruptures of the superior ACLC, which could be assigned to four different tear patterns. Clavicular-sided (AC-1) tears were observed in 46/65 (70.8 %), oblique (AC-2) tears in 12/65 (18.5 %), midportion (AC-3) tears in 3/65 (4.6 %) and acromial-sided (AC-4) tears in 4/65 (6.1 %) of cases. Articular disc size manifestation was significantly (*P* < .001) more pronounced in patients with AC-1 tears (1.89 ± 0.57) compared to patients with AC-2 tears (0.67 ± 0.89). Other cofactors did not influence ACLC tear patterns. ACLC dislocation with incarceration caused mechanical impediment to anatomical ACJ reduction in 14/65 (21.5 %) of cases including all Rockwood-4 dislocations. Avulsion “in continuity” was a consistent mode of failure of the DT fascia. Type-specific operative strategies enabled anatomical ACLC repair of all observed tear types.

**Conclusions:**

Acute ACLC injuries follow distinct tear patterns. There exist clavicular-sided (AC-1), oblique (AC-2), midportion (AC-3) and acromial-sided (AC-4) tears. Articular disc size was a determinant factor of ACLC tear morphology. Mini-open surgery was required in Rockwood-4 and a relevant proportion of Rockwood-5 dislocations to achieve both anatomical ACLC and ACJ reduction. Type-specific operative repair of acute ACLC tears might promote biological healing and lower rates of horizontal ACJ instability following acute ACJ reconstruction.

## Background

In operative treatment of acute acromioclavicular joint (ACJ) dislocations, increasing attention is paid to reconstruction of the superior acromioclavicular ligament complex (ACLC) [2, 10, 12, 25, 26]. The superior ACLC functions as the major horizontal stabilizer of the ACJ [3, 5, 8, 16]. Insufficient superior ACLC healing may contribute to persistent horizontal ACJ instability, which is reported in up to 50 % of cases following both arthroscopic and open reconstruction of acute ACJ dislocations [13, 17, 24, 33]. Horizontal ACJ instability represents a clinically relevant issue, since several studies showed an association with inferior functional outcome [13, 19, 30]. Therefore, modern techniques of acute ACJ reconstruction employ additional synthetic acromioclavicular stabilization consisting of absorbable or non-absorbable high-strength suture cerclages [10, 12, 30]. Biomechanical cadaver studies have proven, that such combined coraco- and acromioclavicular stabilization enables restoration of physiological ACJ stability [2, 26]. However, time-dependent loosening of synthetic acromioclavicular stabilization results in recurrent instability [26]. Hence, definite horizontal ACJ stability will depend on the biomechanical quality of anatomical ACLC healing. Efforts to anatomically repair ACLC injuries might therefore restore horizontal instability in acute ACJ reconstruction [10].

Histological studies showed the acromioclavicular ligaments as a component of the joint capsule being confluent with the articular disc. If present, the articular disc may vary in size and appearance [6, 27]. The deltoid-trapezoidal (DT) fascia inserts on the superior lateral clavicle and superior ACLC providing additional biomechanical reinforcement [31]. Given the close anatomical and biomechanical relationship of the acromioclavicular ligaments, joint capsule, articular disc, and DT fascia insertions, these structures should be regarded as one anatomical unit, which we refer acromioclavicular ligament complex (ACLC). According to a current macroscopic anatomical study of Nakazawa et al. [20], the acromioclavicular ligament consists of a well-developed superoposterior (SP) bundle and a weaker anteroinferior (AI) bundle. In addition to detailed comprehension of biomechanics and anatomy, optimal operative repair also requires profound knowledge of principal characteristics of acute ACLC injuries. However, hardly any clinical findings exist on morphology and influencing cofactors of acute ACLC injuries.

Based on long-term clinical observations, we assumed that distinct and recurrent ACLC tear patterns would exist in acute ACJ dislocations. Another hypothesis was, that specific ACLC tear types would be associated with anatomical characteristics and injuries of related structures (articular disc, DT fascia, bony ACJ morphology), and the underlying type of ACJ dislocation.

Purposes of this prospective, cross-sectional study were 1.) to identify different ACLC tear patterns with development of a differentiated classification system, 2.) to assess prevalences of different ACLC tear patterns, and 3.) to identify influencing cofactors (articular disc, DT fascia, type of ACJ dislocation and bony ACJ morphology).

## Methods

The study was approved by the Ethics Committee of the University of Freiburg (Vote-Nr.: 490/13) and written informed consent was obtained from all participating patients. From 1/2014 to 7/2016, the study consecutively included a total of 65 patients (6 female, 59 male) with complete, acute-traumatic ACJ dislocations type 4 or 5 according to the Rockwood classification [23] treated operatively by means of mini-open ACJ reduction and hook plate stabilization. Mean age was 38.2 years (range, 19–57 years) at the time of surgery. Inclusion criteria were age >18 years, isolated acute-traumatic ACJ dislocation type 4 or 5 and full legal competence. Exclusion criteria were age >60 years, incomplete ACJ dislocations (Rockwood-3A/B), subacute-chronic ACJ dislocations with an interval >3 weeks from trauma to surgery, radiological signs of ACJ degeneration/arthrosis and prior history of ACJ injury or surgery. Indication for surgery was not influenced by the patient’s decision for study participation. During the study period, 74 patients were eligible for study participation corresponding to an inclusion rate of 88 %. Non-inclusion of 9 patients was caused by refusal of study participation (*n* = 3) and incomplete documentation (*n* = 6).

### Preoperative examination

**Fig. 1:**
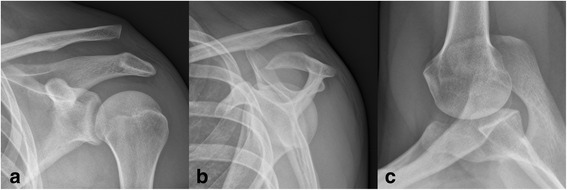
Stress radiography for evaluation of ACJ instability: **a** Anteroposterior stress radiograph shows complete superior separation in a Rockwood-5 injury. **b** Alexander stress view shows complete posterior-superior dislocation in a Rockwood-5 injury. **c** Transaxillary view shows static posterior dislocation in a Rockwood-4 injury

In addition to standardized clinical examination, we performed uniform preoperative radiographic assessment of the shoulder joint and ACJ stability including an anteroposterior (AP) and outlet view of the shoulder joint, bilateral anteroposterior (AP) stress radiographs (Fig. 1a), bilateral Alexander stress views [1] (Fig. 1b) and a transaxillary view (Fig. 1c) of the injured side.

### Operative technique

**Fig. 2:**
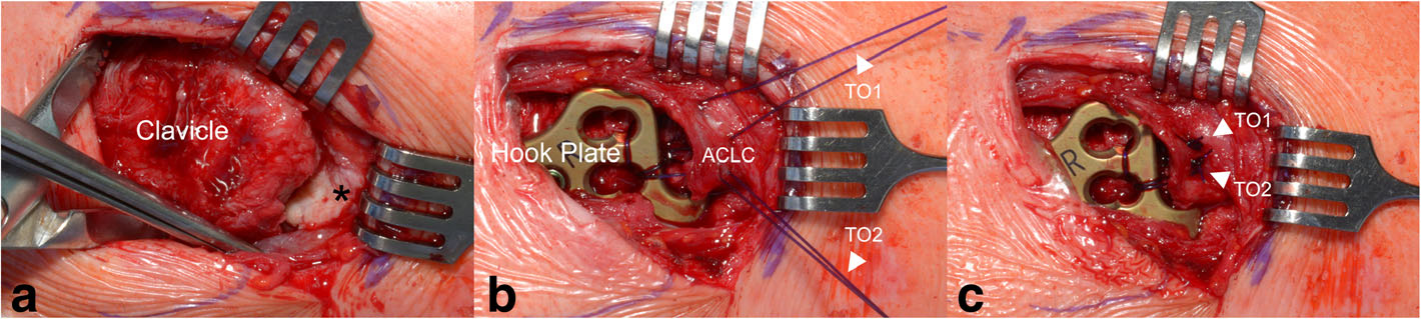
Anatomical repair of a clavicular-sided (AC-1) tear: Superior view of a right AC joint. **a** Typical clavicular-sided ACLC detachment in presence of a meniscoid articular disc (black asterisk). **b** Two transosseous (TO1, TO2), absorbable sutures (white arrowheads) with back-up fixation through lateral holes of the hook plate are placed into the superior ACLC being firmly attached to the acromion. **c** Completed anatomical ACLC repair

Operative technique and intraoperative assessment were performed according to a standardized protocol. Patients were placed supine with 30° of chest elevation. An oblique 4–5 cm (mini-open) saber cut incision was carried out 1 cm medial to the ACJ parallel to the lines of skin cleavage. The DT fascia was identified. After blunt medial and lateral epifascial preparation, the DT fascia was examined for signs of injury (tear, avulsion). Afterwards it was incised along the anterior border of the trapezoid muscle parallel to the longitudinal axis of the lateral clavicle. The DT fascia was separated from the superior ACLC, which was found torn in all cases. The ACLC and ACJ were anatomically reduced under macroscopic and fluoroscopic control. ACJ reduction was temporarily fixed using a 1.8 mm transacromial K-wire. A hook plate (3.5 mm 4-hole LCP Clavicle Hook Plate with 12–18 mm offset, DepuySynthes, West Chester, USA) was implanted for ACJ stabilization. The K-wire was removed. After final fluoroscopic control of anatomical ACJ reduction and correct implant position, anatomical ACLC repair (Fig. 2) was performed using transosseous and/or direct absorbable sutures (MonoPlus, B. Braun Melsungen AG, Melsungen, Germany). Finally, the DT fascia and the wound were closed with absorbable suture material.

### Intraoperative assessment

#### ACLC tear

Meticulous surgical preparation aimed for identification and visualization of the superior ACLC including its entire anterior-posterior and medial-lateral dimensions. All ACLC tears were characterized according to their morphological appearance by the senior observer (DM) and could be clearly assigned to a distinct tear type.

#### Cofactors

Observational analysis of anatomical conditions and concomitant injuries included the articular disc, the DT fascia, underlying type of ACJ dislocation and bony ACJ morphology. Articular disc manifestation was quantified using the size-dependent classification introduced by Salter et al. [27] (0 = absent disc, 1 = remnant disc, 2 = meniscoid disc and 3 = complete disc). Assessment of cofactors included: articular disc dislocation (yes, no), articular disc rupture (yes, no) and injury of the DT fascia (macroscopic tear/discontinuity, insertional avulsion). ACJ dislocations were classified as Rockwood-4 injuries in case of static posterior dislocation and as Rockwood-5 injuries in case of superior dislocation of the lateral clavicle. Bony ACJ morphology was classified in AP radiographs according to three main shape types as described previously by Urist [32] and more recently by Colegate-Stone et al. [4]: vertical (flat), oblique and curved.

### Statistics

Statistical analysis was performed using the software SPSS version 22 (SPSS Inc., Chicago, IL, USA). Descriptive results are given as mean values with ranges or standard deviations (±). The prevalence of the identified ACLC tear types was calculated as percentages of all ACLC injuries. Distributional analysis was performed with Pearson’s Chi-square test. A Mann-Whitney *U* test was used for non-parametric group comparisons. Relative risk calculation served for prognostic assessment of cofactors. With 95 % confidence intervals, statistical significance was assumed for *p* < 0.05.

## Results

All patients showed complete acute-traumatic ACLC disruptions. Each ACLC tear could be clearly assigned to one of four distinct and recurrent tear patterns. Based on these intraoperative observations, we propose a morphological classification of acute ACLC tears.

### Classification and prevalences of acute ACLC tears

#### Clavicular-sided ACLC tear (AC-1)

**Fig. 3:**
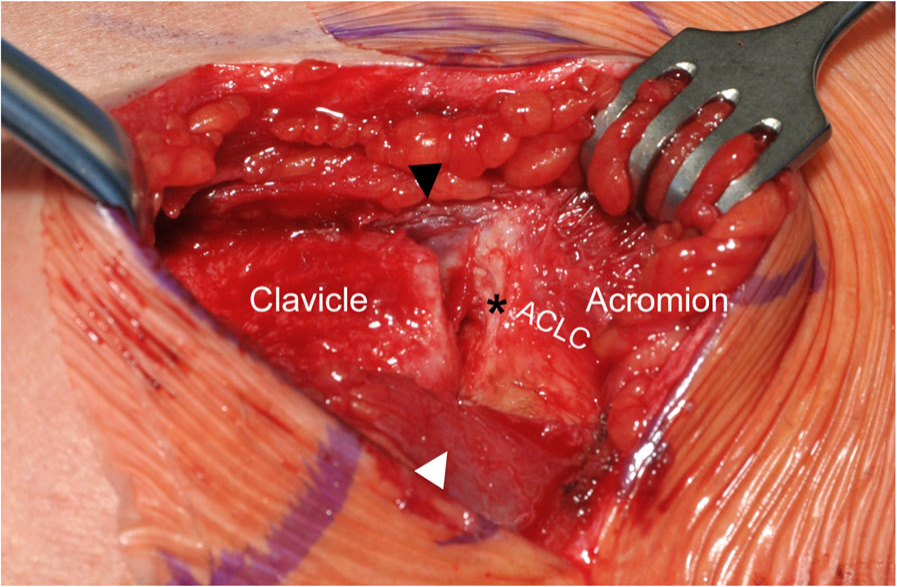
Intraoperative finding of a clavicular-sided (AC-1) tear: Posterior-superior view of a right AC joint. Typical subperiosteal, clavicular-sided ACLC detachment. The meniscoid articular disc (black asterisk) forms an anatomical unit with the superior ACLC showing intact acromial attachment. White arrowhead: trapezoid part of DT fascia, black arrowhead: deltoid part of DT fascia

**Fig. 4:**
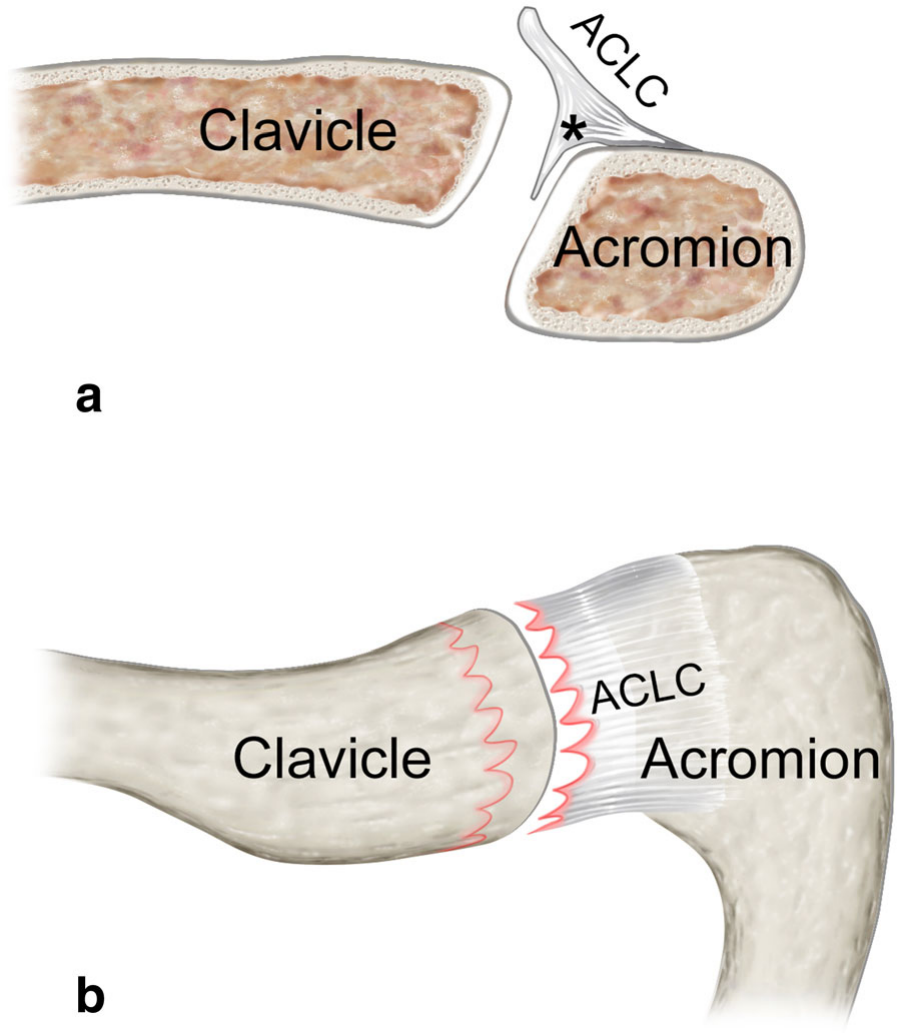
Schematic illustration of a clavicular-sided (AC-1) tear: **a** coronar view and **b** superior view show typical clavicular-sided ACLC detachment. A distinct articular disc (black asterisk) is usually present

Clavicular-sided ACLC detachment (AC-1) involving its entire anterior-posterior dimension was present in 46 (70.8 %) cases. ACLC detachment typically occurred subperiosteally. The lateral aspect of the clavicle showed a pathognomonic “peeled-like” appearance, which we refer to as the “banana sign”. Fig. 3 shows intraoperative findings and Fig. 4 schematically illustrates morphology of a clavicular-sided (AC-1) ACLC tear.

#### Oblique ACLC tear (AC-2)

**Fig. 5:**
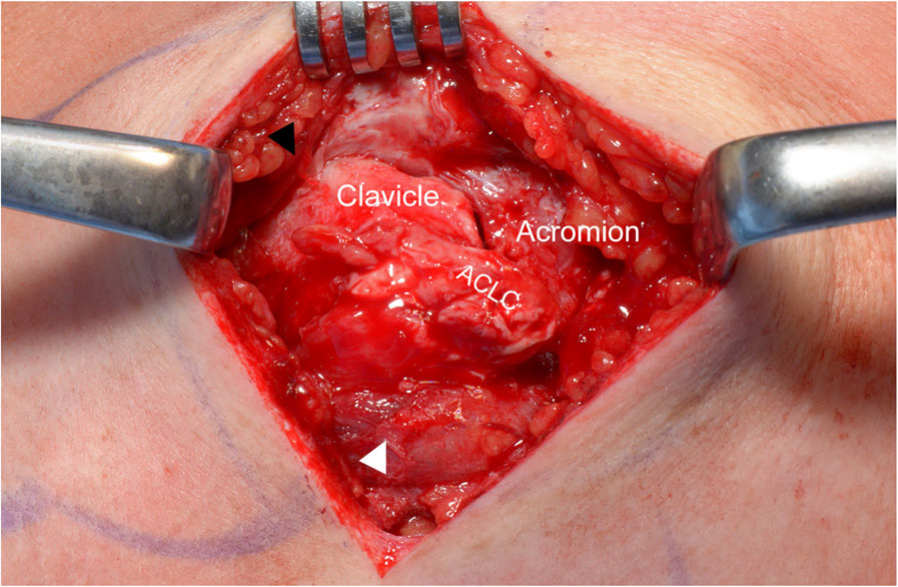
Intraoperative finding of an oblique, anteromedial-posterolateral (AC-2A) tear: Superior view of a right AC joint. Oblique course of ACLC tear extending from anteromedially (clavicularly) throughout the superior ACLC to posterolaterally (acromially). There is no articular disc. White arrowhead: trapezoid part of DT fascia, black arrowhead: deltoid part of DT fascia

**Fig. 6:**
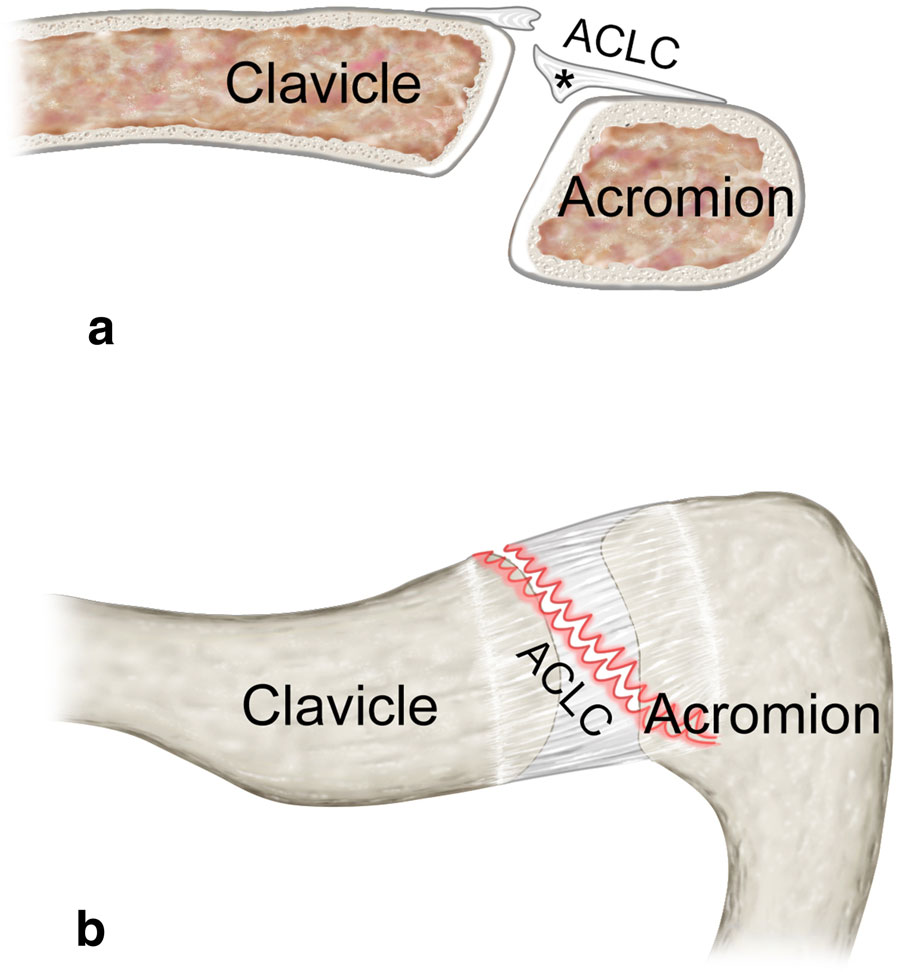
Schematic illustration of an oblique, anteromedial-posterolateral (AC-2A) tear: **a** coronar view and **b** superior view show the oblique course of ACLC tear extending from anteromedially (clavicular) throughout the superior ACLC to posterolaterally (acromial). There is usually no or only a remnant articular disc (black asterisk)

**Fig. 7:**
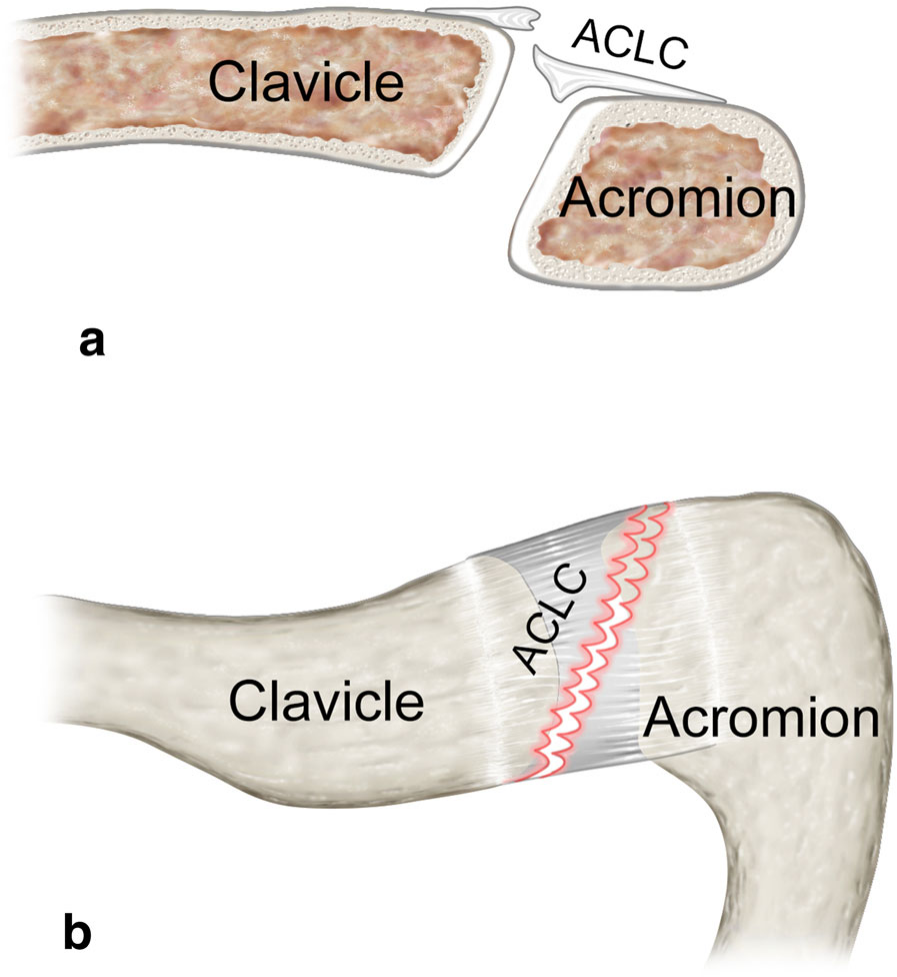
Schematic illustration of an oblique, anterolateral-posteromedial (AC-2B) tear: **a** coronar view and **b** superior view show the oblique course of ACLC tear extending from anterolaterally (acromial) throughout the superior ACLC to posteromedially (clavicular). There is usually no or only a remnant articular disc (black asterisk)

Oblique ACLC tears (AC-2) were found in 12 (18.5 %) patients. According to the course of the tear throughout the superior ACLC, we distinguished two subtypes of oblique tears: an anteromedial-posterolateral (AM-PL) tear (AC-2A) and an anterolateral-posteromedial (AL-PM) tear (AC-2B). AC-2A tears occurred more frequently (*n* = 10) than AC-2B tears (*n* = 2). In anteromedial-posterolateral (AC-2A) tears, the anterior ACLC was detached from the clavicle as observed in clavicular-sided tears. AC-2A tears extended from anteromedially (clavicularly) in an oblique direction throughout the superior ACLC to posterolaterally (acromially). Posteriorly, there was acromial-sided ACLC detachment. Accordingly, anterolateral-posteromedial (AC-2B) tears run from anterolaterally (acromially) to posteromedially (clavicularly) throughout the superior ACLC. Figure 5 shows intraoperative findings and Fig. 6 schematically illustrates morphology of an anteromedial-posterolateral (AC-2A) ACLC tear. Figure 7 schematically illustrates morphology of an anterolateral-posteromedial (AC-2B) ACLC tear.

#### Midportion ACLC tear (AC-3)

**Fig. 8:**
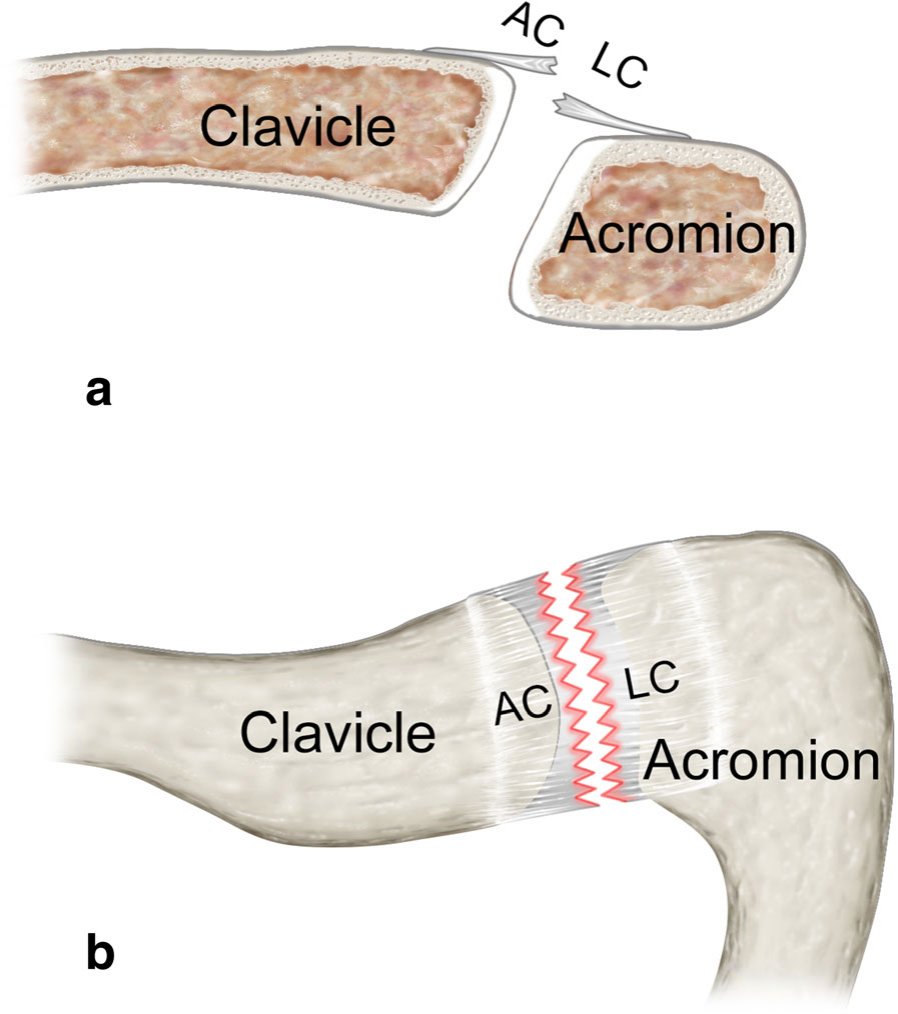
Schematic illustration of a midportion (AC-3) tear: **a** coronar view and **b** superior view show the straight course of ACLC tear running centrally throughout the superior ACLC. There is usually no articular disc

Midportion ACLC tears had occurred in three (4.6 %) patients and proceeded centrally throughout the superior ACLC. Central division of the ACLC resulted in two approximately even parts, each of them remaining firmly attached to the clavicle and acromion, respectively. Figure 8 schematically illustrates morphology of a midportion (AC-3) ACLC tear.

#### Acromial-sided ACLC tear (AC-4)

**Fig. 9:**
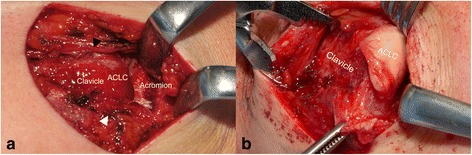
Intraoperative finding of an acromial-sided (AC-4) tear: (A) Superior view and (B) supero-lateral view of a right AC joint. **a** Typical subperiosteal, acromial-sided ACLC detachment. White arrowhead: trapezoid part of DT fascia, black arrowhead: deltoid part of DT fascia. **a** Undersurface of everted superior ACLC without presence of an articular disc

**Fig. 10:**
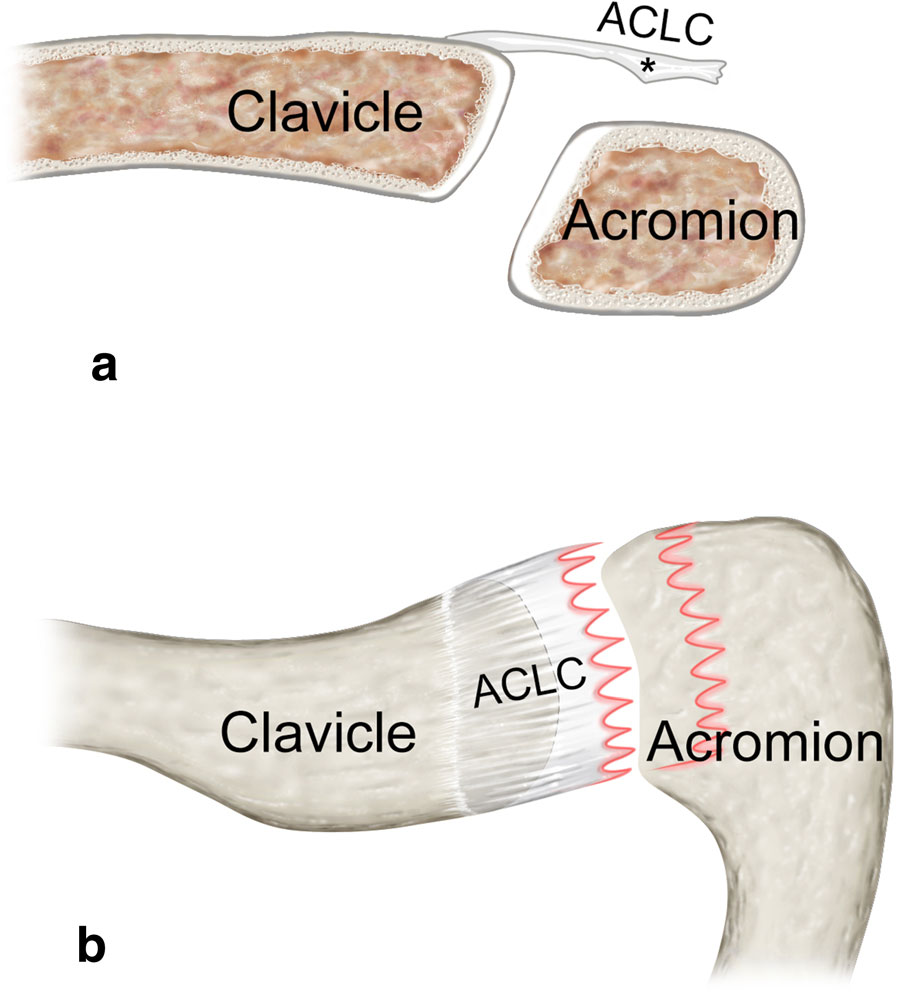
Schematic illustration of an acromial-sided (AC-4) tear: **a** coronar view and **b** superior view show typical acromial-sided ACLC detachment. There is usually no or only a remnant articular disc (black asterisk)

Acromial-sided ACLC detachments (AC-4) were found in four (6.1 %) cases involving its total anterior-posterior dimension. Similarly to clavicular-sided ACLC tears, detachment occurred subperiosteally in all three patients. Figure 9 shows intraoperative findings and Fig. 10 schematically illustrates morphology of an acromial-sided (AC-4) ACLC tear.

Table 1 summarizes the morphological classification of acute ACLC tears and type-specific prevalences.

**Table 1:** Classification and prevalences of acute ACLC tears

ACLC Tear Type		Classification		Prevalence (%)	
Clavicular-sided Tear		AC-1		70.8 %	
Oblique Tear	AM-PL	AC-2	AC-2A	18.5 %	15.4 %
	AL-PM		AC-2B		3.1 %
Midportion Tear		AC-3		4.6 %	
Acromial-sided Tear		AC-4		6.1 %	

### Cofactor analysis

#### Articular disc

A total of 52 (80.0 %) patients presented a definable articular disc. Thereof, the majority of patients (*n* = 37; 56.9 %) had a partial, meniscoid articular disc. Only four (6.1 %) patients showed a complete articular disc. Eleven (16.9 %) individuals presented a small, remnant articular disc, and no disc was found in 13 (20.0 %) cases. Distribution of articular disc sizes was not uniform but varied significantly (*p* < 0.001) between ACLC tear types (Table 2).

**Table 2:** Distribution of articular disc manifestation related to ACLC tear types^a^

		ACLC Tear Type				
		AC-1	AC-2	AC-3	AC-4	Total
Articular Disc Size	0	2	6	3	2	13
	1	4	5	0	2	11
	2	37	0	0	0	37
	3	3	1	0	0	4
	Total	46	12	3	4	65

^a^Pearson’s Chi-square test shows unequal distribution of articular disc size related to ACLC tear types (*P* < .001)

All patients with meniscoid articular discs had clavicular-sided AC-1 tears. Mean size of articular disc manifestation was significantly (*P* < .001) more pronounced in patients with AC-1 tears (1.89 ± 0.57) compared to patients with AC-2 (0.67 ± 0.89). ACLC dislocation with incarceration caused mechanical impediment to anatomical ACJ reduction in 14/65 (21.5 %) of cases. Thereof, articular disc dislocation was found in 12 (18.5 %) individuals. Eleven (16.9 %) patients were diagnosed with articular disc rupture. Thereof, six (9.2 %) patients had sustained additional articular disc dislocation. In those six patients with unstable articular disc dislocation tears, partial discectomy was performed to stabilize the residual intact articular disc and to achieve anatomical ACLC and ACJ reduction. For articular disc injury, there was no statistically significant association with ACLC tear types.

#### Deltoid-trapezoidal fascia

All patients showed injury of the DT fascia in terms of an “in continuity” insertional avulsion. The macroscopic aspect of the DT fascia was thin and elongated. Additional discontinuity presenting as a longitudinal or transverse rupture of <3 cm length was found in ten (15.4 %) patients.

#### Type of ACJ dislocation

The majority of patients (*n* = 57; 87.7 %) were diagnosed with Rockwood-5 injuries showing superior ACJ dislocation. Only 8 (12.3 %) patients showed static posterior ACJ dislocations being consistent with Rockwood-4 injuries. In all patients with Rockwood-4 dislocations and additionally in six patients with Rockwood-5 injuries, the ACLC constituted a mechanical obstacle for anatomical ACJ reduction. The ACLC was found incarcerated into the joint space and mechanically prevented reduction of the lateral clavicle. In these 14 cases, mini-open ACLC visualization and reduction was required to achieve true anatomical ACJ reduction. In subperiosteally avulsed AC-1 tears, the lateral clavicle had to be “reinserted” into the ACLC to obtain anatomical alignment and reduction. Compared to Rockwood-5 injuries, Rockwood-4 dislocations were associated with a 4.1-fold increased relative risk for articular disc rupture, a 7.1-fold increased relative risk for articular disc dislocation and a 9.5-fold increased relative risk for necessity of mini-open ACJ reduction due to ACLC incarceration. With regard to the DT fascia, 6/8 patients with Rockwood-4 dislocations showed additional longitudinal tears of <3 cm length of the posterior trapezoid part. Thereof, only one patient presented with a posterior perforation of the lateral clavicle throughout the DT fascia. In contrast, only 4/57 (7.2 %) patients with a Rockwood-5 injury had sustained an additional transverse tear of <3 cm length running parallel to the joint line. Compared to Rockwood-5 injuries, Rockwood-4 dislocations involved a 10.7-fold increased risk for rupture of the DT fascia.

#### Bony ACJ morphology

A vertical (flat) shape of the ACJ was found in 22/65 (33.9 %) patients, an oblique one in 35/65 (53.8 %) patients and a curved one in 8/65 (12.3 %) patients. However, there was no statistically significant association with ACLC tear patterns (*p* = 0.42).

### Anatomical ACLC repair

**Fig. 11:**
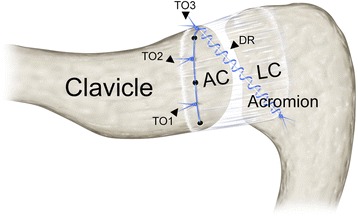
Schematic illustration of anatomical ACLC repair: AC-1 repair with two transosseous sutures (TO1, TO2). AC-2A repair with a transosseous suture (TO3) and direct repair (DR) with a running stitch. Anatomical AC-4 and AC-2B repair can be achieved applying the same principles

Type-specific approaches enabled anatomical operative ACLC repair of all observed tear patterns. Figure 3 exemplifies anatomical repair of an AC-1 tear using a transosseous, absorbable suture. Based on current findings, we suggest type-specific primary ACLC repair (Fig. 11). Clavicular-sided AC-1 and acromial-sided AC-4 tears can be repaired using transosseous sutures or mini suture anchors. Repair of oblique AC-2 tears can be achieved combining transosseous and direct sutures. Midportion AC-3 tears are suited for direct suture repair.

## Discussion

This study is the first to systematically investigate and describe ACLC tear patterns in acute ACJ dislocations, and hereby provides fundamental knowledge of characteristics of acute ACLC injuries. Using a novel morphological classification system, acute ACLC disruptions could be assigned to clavicular-sided (AC-1), oblique (AC-2), midportion (AC-3), and acromial-sided (AC-4) tears. Prevalences of specific ACLC tear types (AC-1-4) depended on the size of the articular disc. In accordance with Salter et al. [27], the majority of our patients (57 %) had a meniscoid (partial) articular disc. A proportion of 37 % showed no articular disc or only remnants of an articular disc. Histological studies have confirmed that the articular disc is confluent with the superior capsular-ligamentous structures forming an anatomical-biomechanical unit [6, 27]. Hereby, the articular disc seems to function as a biomechanical reinforcement of the superior ACLC. Recently, Nakazawa et al. [20] identified a strong superoposterior (SP) bundle and a more delicate anteroinferior (AI) bundle of the acromioclavicular ligament. The articular disc predominantly attaches to the supero-lateral (acromial) SP bundle shifting its biomechanical point of weakness to the clavicular insertion zone [6, 20].

These principal anatomical-biomechanical aspects strongly support our observations on acute ACLC tear morphotypes and their correlation to the size of articular disc manifestation. In acute-traumatic ACJ dislocation, presence of a pronounced articular disc predominantly induced a clavicular-sided (AC-1) tear due to increased stress shielding of the supero-lateral ACLC. ACLC detachment typically occurred subperiosteally, since bony insertion zones are biomechanically augmented by fibrocartilaginous entheses. Clinically, such subperiosteal clavicular-sided ACLC avulsion causes a pathognomonic “peeled-like” appearance, which we refer to as the “banana sign”. In case of a remnant or absent articular discs, the central and lateral portions of the superior ACLC became vulnerable to rupture. Accordingly, oblique and acromial-sided tears were associated with smaller articular disc sizes. Midportion tears were rare and only occurred in case of complete absence of the articular disc. Thus, the central superior ACLC in between fibrocartilaginous insertion zones represented the “weakest link of the chain”. Interestingly, bony ACJ morphology did not influence ACLC tear patterns. In addition to the size of articular disc manifestation, biomechanics of trauma is likely to substantially influence ACLC tear patterns, particularly with regard to the direction of translation (force vector) of the acromion related to the clavicle. In this context, principal biomechanical studies should follow.

To date, hardly any knowledge exists about the morphology of acute ACLC tears, and to our best knowledge no previous study focused on this topic. Rockwood et al. [23] described different degrees of acute ACLC injuries from sprain to complete rupture but did not analyze the morphology of ACLC tears. Hessmann et al. [9] performed AC ligament repair and augmentation in acute type 3, 4 and 5 injuries. They usually found the ACLC to be “avulsed from the lateral clavicle” without describing other tear types. In a biomechanical cadaver study, Freedman et al. [7] observed midsubstance ACLC tears resulting from superior translation of the clavicle. Present study entails important implications for acute ACJ reconstruction and operative ACLC repair. Specific types of ACLC tears were not related to different types of ACJ dislocation. However, a Rockwood-4 injury represented a prognostic risk factor for articular disc dislocation/rupture and ACLC incarceration. Fourteen (21.5 %) of patients (all Rockwood-4 and 10.5 % of Rockwood-5 dislocations) required mini-open surgery to obtain anatomical ACLC and ACJ reduction. In these cases, ACLC incarceration caused mechanical obstruction. Attempts of closed reduction probably would have caused non-anatomical ACJ and ACLC reduction, potentially resulting in insufficient ACLC healing and persistent horizontal ACJ instability. According to our experience, the articular disc can be preserved in majority of acute patients. We performed partial discectomy in only six (9.2 %) patients with unstable articular disc dislocation tears to obtain a stable residual articular disc. Remaining unstable tears might have a negative impact on outcome. With regard to the DT fascia, avulsion “in continuity” represented a consistent mode of failure. Insertional avulsion and thinning of the DT fascia were found in both Rockwood− 4 and −5 dislocations, whereas additional tears usually occurred in type 4 dislocations only. These longitudinal or transverse tears were rather small (<3 cm) and could be easily repaired. Perforation of the lateral clavicle throughout the DT fascia was previously considered as the pathoanatomical correlate of Rockwood-4 injuries. However, clavicular perforation was found in only one case, and therefore has to be regarded as an exceptional pattern of injury.

**Fig. 12:**
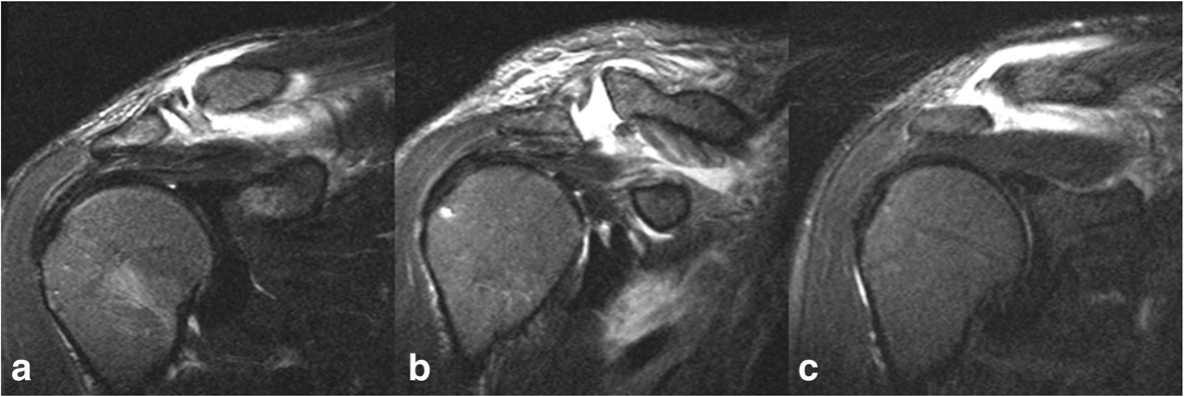
Exemplary ACJ-specific MRIs of ACLC tear types: **a** AC-1 tear with complete articular disc, **b** AC-1 tear with remnant articular disc and **c** AC-4 tear without articular disc manifestation

Current pathoanatomical findings substantially expand understanding of acute ACJ dislocations and significantly influence their operative treatment. This study showed, that complex ACLC injuries with incarceration rather than injury of the DT fascia prevent anatomical ACJ reduction. From our point of view, mini-open ACLC and ACJ reduction should be performed in Rockwood-4 injuries with true static posterior ACJ dislocation. Moreover, a relevant proportion of about 10 % of Rockwood-5 dislocations may also require mini-open reduction. At the beginning of the procedure, surgeons should carefully verify feasibility of anatomical ACJ reduction prior to application of closed techniques of ACJ reduction and stabilization. Any fluoroscopic evidence of joint space enlargement suggests presence of ACLC incarceration as an obstacle to reduction. Thus, both the surgeon and the patient should be prepared for conversion to a mini-open procedure. In this context, preoperative MRI could prove of value for assessment ACLC tear patterns and identification of patients benefiting from mini-open surgery due to otherwise limited healing potential. Current ACJ-specific MRI techniques allow detailed assessment of ligamentous structures including the articular disc [11, 21, 29]. In our own series, we were able to distinguish distinct ACLC tear types and to diagnose articular disc dislocations in selective cases by means of ACJ-specific MRI (Fig. 12). However, future studies should evaluate the role of preoperative MRI for diagnosis of acute ACLC injuries.

Several studies have shown significant association of horizontal instability with inferior clinical outcome following arthroscopically-assisted and open acute ACJ reconstruction [13, 19, 30]. Scheibel et al. [30] reported inferior Taft (9.2 versus 11.4. points) and ACJI scores (92.3 versus 63.3 points) compared to patients without residual horizontal instability following double TightRope ACJ reconstruction. Jensen et al. [13] found considerable impairment of not only ACJ-specific but also glenohumeral function (normalized Constant score [15]: 76 % versus 93 %) for both double TightRope and hook plate stabilization. Thus, insufficient ACLC healing causing persistent horizontal ACJ instability represents a clinically relevant issue. Beginning from the 1980s, ACLC suturing was an important component of acute ACJ stabilization using tension band wiring, screws or Balser (hook) plates [14, 18, 22]. However, none of the techniques succeeded substantially due to hardware complications, iatrogenic fractures, persistent ACJ instability and ACJ arthrosis/synostosis resulting from non-physiological ACJ kinematics. Biomechanical studies demonstrated that only combined, non-rigid coraco- and acromioclavicular stabilization may restore physiological ACJ stability [2, 26]. Saier et al. [26] employed an x-shaped acromioclavicular cerclage consisting of high strength suture tapes. Nonetheless, final horizontal ACJ stability will be determined by biomechanical quality of biologic ACLC healing due to the likelihood of time-dependent loosening/failure of synthetic stabilization material. Optimal biomechanical ACLC healing requires both anatomical ACJ/ACLC reduction and physiological ACJ stabilization [10]. Present study showed, that mini-open operative ACLC treatment was required in 21.5 % of acute ACJ dislocations to achieve anatomical reduction. This finding might at least partly explain the high rates of horizontal ACJ instability following closed techniques of acute ACJ reconstruction [13, 17, 24, 28, 30, 33]. Additional operative ACLC repair might support anatomical ACLC healing, if sufficient biomechanical augmentation is provided during the phase of healing [10]. In this context, we propose type-specific operative techniques for anatomical ACLC repair (Fig. 11).

Limiting, the superior surgical approach allowed accurate inspection and assessment of anterior, superior and posterior except inferior ACLC portions. Therefore, inferior ACLC tear morphology might vary from superior ACLC tear types. However, this limitation does not substantially affect significance of present study, since the superior ACLC represents the major restraint to posterior dislocation, and therefore clinical efforts focus on superior ACLC repair [2, 5, 10, 12, 16]. Analysis of clinical and radiological outcome was not aim of this cross-sectional study but should be subject of future comparative clinical trials.

## Conclusion

There exist distinct and recurrent morphotypes of ACLC tears in acute ACJ dislocations. Clavicular-sided (AC-1) tears were observed in 72 %, oblique (AC-2) tears in 19 %, midportion (AC-3) tears in 4 % and acromial-sided (AC-4) tears in 5 %. Morphology of acute ACLC tears depended on anatomical conditions of the acromioclavicular ligament complex (ACLC), particularly on the size of the articular disc. AC-1 tears usually occur in presence of a pronounced articular disc, whereas AC-2-AC-4 tears are associated with a remnant or absent articular disc. Acute ACLC with dislocation and incarceration caused mechanical obstruction to anatomical ACJ reduction in 21.5 % of cases including all Rockwood-4 dislocations. In these cases, mini-open ACLC reduction was required to achieve anatomical ACLC and ACJ reduction. With regard to the DT fascia, avulsion “in continuity” represented the consistent mode of failure. Type-specific operative strategies enabled anatomical operative ACLC repair of all observed tear patterns. Prospective clinical studies should evaluate clinical implications of operative ACLC and whether combined coraco-, acromioclavicular stabilization and anatomical ACLC repair is able to diminish rates of horizontal ACJ instability in acute ACJ reconstruction.

## Abbreviations

AC: Acromioclavicular
ACJ: Acromioclavicular joint
ACLC: Acromioclavicular ligament complex
AL-PM: Anterolateral-posteromedial
AM-PL: Anteromedial-posterolateral
DT: Deltoid-trapezoidal
TO: Transosseous
